# Supplementary material for: Effectiveness and Safety of Ayurvedic Medicines in Type 2 Diabetes Mellitus Management: A Systematic Review and Meta-Analysis
Source: Front Pharmacol. 2022 Jun 8;13:821810. doi: 10.3389/fphar.2022.821810 (PMC9213670; doi:10.3389/fphar.2022.821810)
Supplement: Supplementary file 1 [file DataSheet1.zip › Supplementary Material/Forest and Funnel Plots/Enicostemma axillare (versus OAD)/PPBG.pdf]

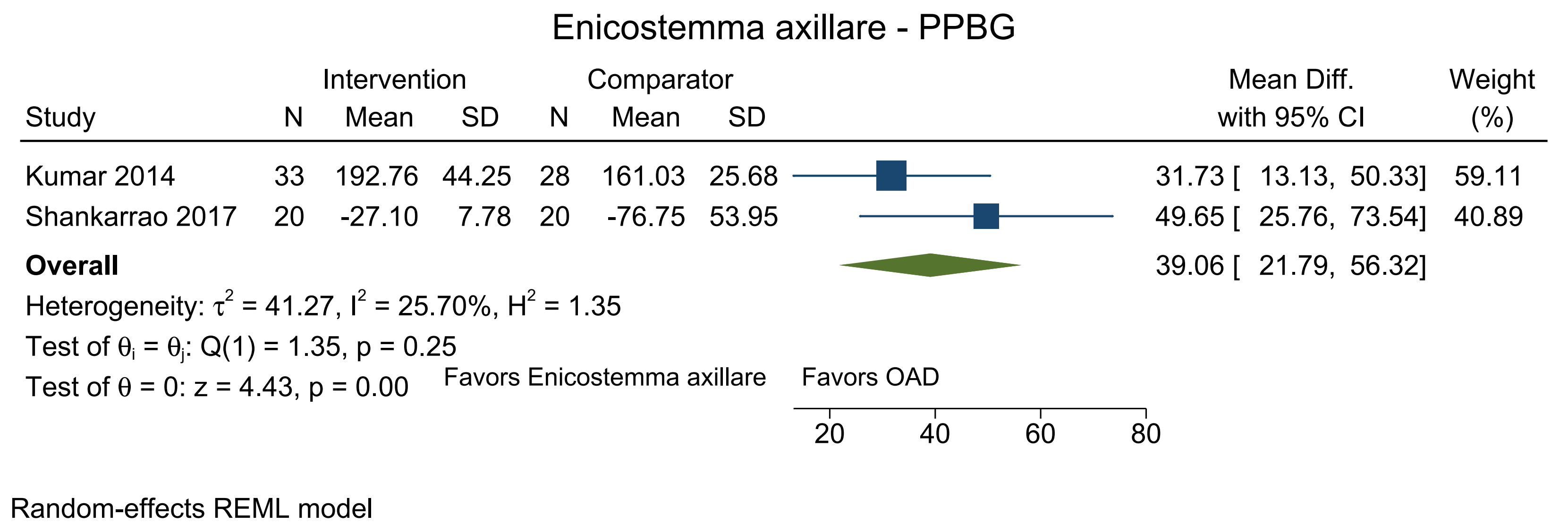
Enicostemma axillare - PPBG
Intervention
Comparator
Study
N
Mean
SD
N
Mean
SD
Mean Diff. with 95% CI
Weight (%)
Kumar 2014 33 192.76 44.25 28 161.03 25.68 31.73 [13.13, 50.33] 59.11
Shankarrao 2017 20 -27.10 7.78 20 -76.75 53.95 49.65 [25.76, 73.54] 40.89
Overall 39.06 [21.79, 56.32]
Heterogeneity: τ² = 41.27, I² = 25.70%, H² = 1.35
Test of θi = θj: Q(1) = 1.35, p = 0.25
Test of θ = 0: z = 4.43, p = 0.00
Favors Enicostemma axillare
Favors OAD
20
40
60
80
Random-effects REML model
